# Evaluation of a saliva molecular point of care for the detection of SARS-CoV-2 in ambulatory care

**DOI:** 10.1038/s41598-021-00560-8

**Published:** 2021-10-26

**Authors:** Jérôme LeGoff, Solen Kernéis, Caroline Elie, Séverine Mercier-Delarue, Nabil Gastli, Laure Choupeaux, Jacques Fourgeaud, Marie-Laure Alby, Pierre Quentin, Juliette Pavie, Patricia Brazille, Marie Laure Néré, Marine Minier, Audrey Gabassi, Chrystel Leroy, Béatrice Parfait, Jean-Marc Tréluyer, Constance Delaugerre

**Affiliations:** 1 Virologie, AP-HP, Hôpital Saint Louis, 1 Avenue Claude Vellefaux, 75010 Paris, France; 2 INSERM, Equipe INSIGHT, U976, 75010, Université de Paris, Paris, France; 3 INSERM, IAME, Université de Paris, 75018 Paris, France; 4 Equipe de Prévention du Risque Infectieux, AP-HP, Hôpital Bichat, 75018 Paris, France; 5 Institut Pasteur, Epidemiology and Modelling of Antibiotic Evasion, 75015 Paris, France; 6 Clinical Research Unit / Clinical Investigation Center, APHP, Necker-Enfants Malades Hospital, 75015 Paris, France; 7 EA 7323 Pharmacologie et Thérapeutique de l’enfant et de la Femme Enceinte, Université de Paris, 75015 Paris, France; 8 Plateforme Covid IDF, AP-HP Centre, 75014 Paris, France; 9 Virologie, AP-HP, Hôpital Necker-Enfants Malades, 75015 Paris, France; 10 Centre de Dépistage COVISAN 13 14 15, Communauté Professionnelle de Territoire de Santé, 75014 Paris, France; 11 Immuno-Infectiologie, AP-HP, Hôtel Dieu, 75004 Paris, France; 12 Centre de Dépistage COVISAN, AP-HP, Hôtel-Dieu, 75004 Paris, France; 13 Centre de Ressources Biologiques, Hôpital Cochin, 75014 Paris, France; 14 Plateforme SeqOIA, AP-HP, 75014 Paris, France; 15 INSERM, U944, 75010, Université de Paris, Paris, France

**Keywords:** Virology, SARS-CoV-2, Molecular medicine, Infectious diseases

## Abstract

Rapid identification of SARS-CoV-2-infected individuals is a cornerstone for the control of virus spread. The sensitivity of SARS-CoV-2 RNA detection by RT-PCR is similar in saliva and nasopharyngeal swabs. Rapid molecular point-of-care tests in saliva could facilitate, broaden and speed up the diagnosis. We conducted a prospective study in two community COVID-19 screening centers to evaluate the performances of a CE-marked RT-LAMP assay (EasyCoV) designed for the detection of SARS-CoV2 RNA from fresh saliva samples, compared to nasopharyngeal RT-PCR, to saliva RT-PCR and to nasopharyngeal antigen testing. Overall, 117 of the 1718 participants (7%) tested positive with nasopharyngeal RT-PCR. Compared to nasopharyngeal RT-PCR, the sensitivity and specificity of the RT-LAMP assay in saliva were 34% and 97%, respectively. The Ct values of nasopharyngeal RT-PCR were significantly lower in the 40 true positive subjects with saliva RT-LAMP (Ct 25.9) than in the 48 false negative subjects with saliva RT-LAMP (Ct 28.4) (*p* = 0.028). Considering six alternate criteria for reference tests, including saliva RT-PCR and nasopharyngeal antigen, the sensitivity of saliva RT-LAMP ranged between 27 and 44%. The detection of SARS-CoV-2 in crude saliva samples with an RT-LAMP assay had a lower sensitivity than nasopharyngeal RT-PCR, saliva RT-PCR and nasopharyngeal antigen testing.

*Registration number*: NCT04578509.

## Introduction

The coronavirus disease 2019 (COVID-19) pandemic has had a significant impact on the healthcare system and socioeconomic activity. Early diagnosis is critical for prompt actions regarding patient management, infection control and public health control measures^1^. Since transmission can occur from asymptomatic or pre-symptomatic patients, mass testing, together with rigorous contact tracing and isolation, has been recommended to control the pandemic^2–4^. This strategy implies rapid and reliable testing methods. Although molecular detection of SARS-CoV-2 RNA in nasopharyngeal swabs is considered the “gold standard” for identifying infected individuals^1,5^, nasopharyngeal sampling requires specific sampling equipment and trained personnel and may be difficult in some patients. Mass RT-PCR testing is carried out in specialized laboratories and requires several hours before results are released. Altogether, these constraints limit access to massive testing, increase time-to-result and consequently delay the isolation of contagious individuals^6^.

Rapid antigen point-of-care (Ag) testing allows the drawback of RT-PCR time-to-result to be overcome but still requires nasopharyngeal sampling. The sensitivity of Ag tests was estimated at 50%-90% and specificity at 90–100% compared to nasopharyngeal RT-PCR^7,8^. Recently, anterior nasal self-sampling has been tested to reduce patient discomfort and avoid requirements for nasopharyngeal swabbing^9,10^.

Self-collected saliva is non-invasive and easy to collect and thus more suitable for mass screening than nasopharyngeal sampling^11–14^. Recent meta-analyses assessed the performances of saliva RT-PCR tests for the diagnosis of COVID-19^15–18^, and we recently confirmed in a large prospective study the excellent sensitivity of saliva RT-PCR, as compared to nasopharyngeal RT-PCR, for the detection of SARS-CoV-2 in community screening centers^19^.

The combination of saliva sampling with rapid point-of-care testing could facilitate screening and the isolation of infected individuals. Rapid single-use RT-PCR assays for SARS-CoV-2 RNA detection are available but were validated mainly on nasopharyngeal samples^20,21^ and rarely in saliva^22^; furthermore, they require sophisticated equipment and remain expensive. Nucleic acid detection based on isothermal amplification, such as loop-mediated isothermal amplification (LAMP), are interesting approaches, as they simplify analytical processes, reduce costs and enable faster diagnoses. The sensitivity of RT-LAMP directly from nasopharyngeal swabs (NPS) samples varies from 65 to 87% compared to RT-PCR. A few studies have tested RT-LAMP on self-collected saliva without RNA extraction. Sensitivities ranged from 45% to 85%, results being better after RNA purification than from crude samples^23–26^. No studies have estimated the performances of RT-LAMP on saliva samples as point-of-care systems directly in screening centers.

We conducted a prospective study in two community COVID-19 screening centers to evaluate the performances of a CE-marked RT-LAMP assay specifically designed for the detection of SARS-CoV-2 RNA from fresh saliva samples compared to nasopharyngeal RT-PCR, saliva RT-PCR and nasopharyngeal Ag tests.

## Results

### Participants

**Figure 1:**
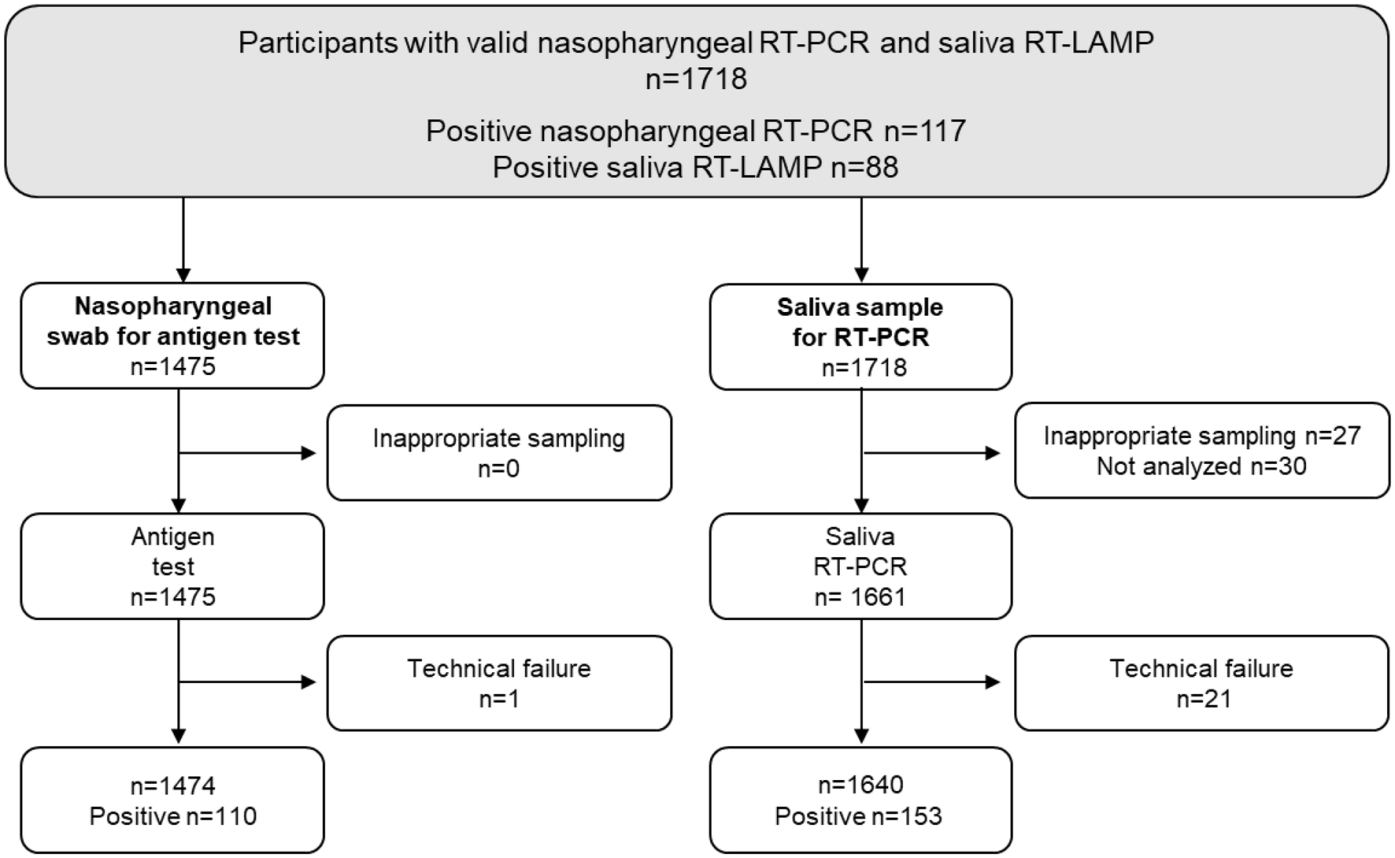
Study flowchart.

Between November 4, 2020, and February 15, 2021, 1718 participants were enrolled with nasopagryngeal sampling for RT-PCR and saliva sampling for RT-LAMP assay. Details of the samples collected and tests performed for the nasopharyngeal antigen assay and saliva RT-PCR are presented in Fig. 1. The median age of study participants was 37 years [26–52] and 55% were females (Table 1). Indications for testing and clinical symptoms reported on the day of inclusion are detailed in Table 1. One to three symptoms were observed in 530/1712 (31%) participants.

**Table 1:** Characteristics of study participants. Results are presented as N (%) or median [interquartile range].

	Total N = 1718
Age, years	37 [26–52]
Females	944 (55)
Contact with a confirmed case	548 (32)
Time from last contact, days	6 [1–7]
Presence of symptoms on day of testing	691 (40)
Time from symptom onset, days	3 [2–4]
Cough	329 (19)
Headaches	268 (16)
Runny nose	264 (15)
Asthenia	215 (13)
Muscle pain	192 (11)
Fever	163 (9)
Diarrhea	107 (6)
Chills	88 (5)
Anosmia	46 (3)
Shortness of breath	53 (3)
Chest pain	50 (3)
Smoking in the last 24 h	331 (19)
Consumption of alcohol in the last 24 h	371 (22)
Consumption of coffee in the last hour	347 (20)
Tooth brushing in the last 2 h	736 (43)
Mouth washing in the last 2 h	61 (4)

### SARS-CoV-2 positive results

Overall, 117/1718 (7%) tested positive on nasopharyngeal RT-PCR: 78/691 (11%) in symptomatic and 39/1027 (4%) in asymptomatic participants (Table 2). Detection rates were 2%, 9% and 7% for saliva RT-LAMP, saliva RT-PCR and nasopharyngeal Ag test, respectively.

**Table 2:** Number of positive samples according to the technical procedure: nasopharyngeal RT-PCR, saliva RT-LAMP or RT-PCR and nasopharyngeal antigen test.

	Positive/total (%)	Presence of symptoms on day of testing	
		Symptoms	No symptoms
Nasopharyngeal RT-PCR	117/1718 (7%)	78/691 (11%)	39/1027 (4%)
Saliva RT-LAMP	88/1718 (5%)	47/691 (7%)	41/1027 (4%)
Saliva RT-PCR	153/1640 (9%)	93/662 (14%)	60/978 (6%)
Nasopharyngeal antigen test	110/1474 (7%)	78/652 (12%)	32/822 (4%)

### Performance of the detection of SARS-CoV-2 infection

**Figure 2:**
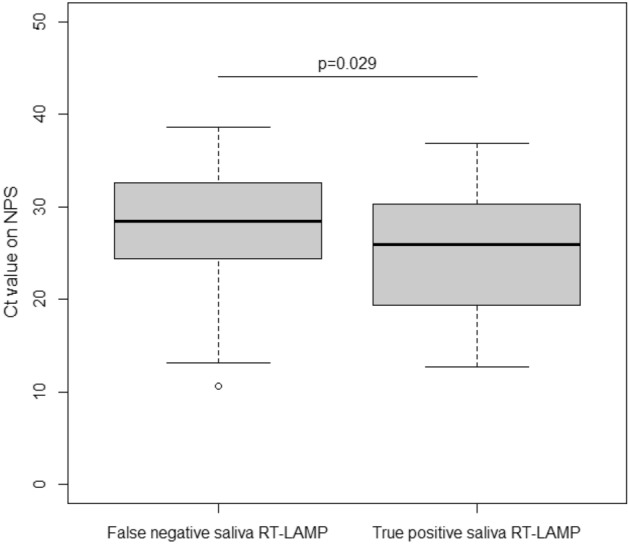
Nasopharyngeal SARS-CoV-2 RT-PCR Ct values according to saliva RT-LAMP results. Ct values of SARS-CoV-2 RT-PCR on nasopharyngeal samples (NPS) in individuals with saliva samples negative with the RT-LAMP assay (False negative) and those with saliva samples positive with the RT-LAMP assay (True positive) are presented in boxplots.

The diagnostic accuracy of the two methods on saliva and the nasopharyngeal Ag test are presented in Table 3. Compared to RT-PCR on NPS, the sensitivity of saliva RT-LAMP was 34% (95% Confidence Interval (95%CI): 26–44). The sensitivity of saliva RT-PCR was 93% (95%CI: 86–97) and that of nasopharyngeal Ag test was 85% (95%CI: 77–91). The sensitivity and specificity of saliva RT-LAMP were similar in symptomatic and asymptomatic participants. Sensitivity analyses of saliva RT-LAMP according to six references (Table 4) showed similar results to the main analysis. The sensitivities of saliva RT-LAMP ranged between 27 and 44%. Its sensitivity was 37% (95%CI: 28–47%) compared to nasopharyngeal antigen test, and 30% (95%CI: 23–38) compared to saliva RT-PCR. The saliva RT-LAMP sensitivity was 40% (95%CI: 28–53) for cycle threshold (Ct) values less than or equal to 28 and 26% (95%CI: 15–40%) for Ct values greater than 28. As displayed in Fig. 2, the Ct values of nasopharyngeal RT-PCR were significantly lower in the 40 true positive subjects with saliva RT-LAMP (25.9 [19.4–30.2]) than in the 48 false negative subjects with saliva RT-LAMP (28.4 [24.4–32.6], p = 0.028), with nasopharyngeal RT-PCR serving as the reference test.

**Table 3:** Diagnostic accuracy of the saliva RT-LAMP or RT-PCR and the nasopharyngeal antigen test as compared to the reference standard (nasopharyngeal RT-PCR, positivity defined as at least one target gene detected), according to the presence of symptoms in study participants.

	Total, n	Positive samples, N	Sensitivity (95% CI)	Specificity (95% CI)
Saliva RT-LAMP	1718	40	34% (26–44)	97% (96–98)
Symptoms	691	25	32% (22–44)	96% (95–98)
No symptoms	1027	15	38% (23–55)	97% (96–98)
Saliva RT-PCR	1640	153	93% (86–97)	97% (96–97)
Symptoms	662	93	93% (85–98)	96% (94–97)
No symptoms	978	60	92% (78–98)	97% (96–98)
Nasopharyngeal antigen test	1474	110	85% (77–91)	99% (98–99)
Symptomsss	652	78	90% (81–96)	98% (96–99)
No symptoms	822	32	74% (57–88)	99% (98–100)

*95% CI* 95% Confidence Interval.

**Table 4:** Sensitivity analysis of diagnostic accuracy of the saliva RT-LAMP test, as compared to several references.

Reference standard	Total, n	Positive samples, N	Sensitivity (95% CI)	Specificity (95% CI)
NPS RT-PCR ≥ 2 targets	1718	88	35% (26–45)	97% (96–98)
Symptoms	691	47	34% (24–46)	96% (95–98)
No symptoms	1027	41	36% (21–54)	97% (96–98)
NPS RT-PCR ≥ 1 target and Ct value < 32	1718	88	37% (27–47)	97% (96–98)
Symptoms	691	47	36% (24–49)	96% (94–98)
No symptoms	1027	41	38% (22–56)	97% (96–98)
Saliva RT-PCR ≥ 1 target	1640	85	30% (23–38)	97% (96–98)
Symptoms	662	45	28% (19–38)	96% (95–98)
No symptoms	978	40	33% (22–47)	98% (97–99)
NPS RT-PCR ≥ 1 target or Saliva RT-PCR ≥ 1 target	1648	87	28% (22–36)	97% (96–98)
Symptoms	667	47	27% (19–37)	97% (95–98)
No symptoms	981	40	30% (20–43	98% (97–99)
NPS RT-PCR ≥ 1 target or Saliva RT-PCR ≥ 1 target and Ct value < 32	1646	87	34% (26–42)	97% (96–98)
Symptoms	666	47	30% (21–41)	97% (95–98)
No symptoms	980	40	40% (26–55)	98% (97–99)
NPS antigen	1474	79	37% (28–47)	97% (96–98)
Symptoms	652	45	35% (24–46)	97% (95–98)
No symptoms	822	34	44% (26–62)	97% (96–98)

*95% CI* 95% confidence interval.

## Discussion

In this large prospective controlled study, the performance of a rapid RT-LAMP assay performed with crude saliva samples directly after saliva collection was analyzed. We used a CE-marked assay specifically designed for saliva samples and for point-of-care use. The test was authorized in France in November 2020 in symptomatic individuals for whom nasopharyngeal sampling was impossible or difficult. Our results demonstrated, in a rigorous real-life evaluation, a low sensitivity of this method (34%) compared to nasopharyngeal RT-PCR. Its sensitivity remained low regardless of the reference test considered (saliva RT-PCR, nasopharyngeal Ag test) and with or without symptoms, ranging between 27 and 44%.

Our results differed greatly from the sensitivity of 86% (95CI 78%-94%) reported in Santos Schneider et al.’s study^27^, and regardless of the reference test used, nasopharyngeal RT-PCR or any other composite reference test including saliva RT-PCR and antigen test. In Santos Schneider et al.’s study, the authors evaluated the EasyCOV® assay in a central laboratory and tested each sample in triplicate. A sample was considered positive if at least two replicates out of three were positive. In our study, we tested all samples once directly in screening centers and according to manufacturer instructions, and according to its expected use in routine conditions.

No difference in RT-LAMP sensitivity was reported between symptomatic and asymptomatic participants. The median time of testing was 3 days after symptom onset or 6 days after last contact of confirmed case. In 103 subjects already diagnosed for COVID-19, Nagura-Ikeda et al. reported, with another RT-LAMP assay, sensitivity results on saliva that differed according to the clinical state^23^. The RT-LAMP assay was performed with nucleic acid saliva extracts instead of crude saliva. Overall sensitivity was 71% compared to the nasopharyngeal RT-PCR, with a higher sensitivity (85%) in patients tested within 9 days of symptom onset than after 9 days (44%). In asymptomatic individuals, the sensitivity was 60%.

According to other studies evaluating RT-LAMP tests, the critical step for sensitivity seemed to be RNA extraction^13,23,25,28^. A high level of concordance between RT-PCR on nasopharyngeal samples and RT-LAMP on saliva was observed when an automated extraction step was used. In a limited series of 34 positive samples (17 nasopharyngeal swabs and 17 saliva) tested by RT-PCR, Taki et al. reported the sensitivity of an RT-fluorescence LAMP assay performed with nucleic acid extracts as being 97% and 100% in nasopharyngeal and saliva samples, respectively^25^. Without RNA extraction on the same samples, sensitivities decreased to 71% and 47%, respectively, suggesting that the RNA extraction process may be critical for SARS-CoV-2 RNA detection by RT-LAMP, especially for saliva samples. In our study, the RT-LAMP assay is an extraction-free test with 10 min of heating at 80 °C for virus inactivation and viral RNA release. This quick step suitable for a point-of-care test might be not optimal for RT-LAMP reactions with saliva samples, and results may depend on miscellaneous factors depending on the quality of saliva (volume, pH, viscosity, food by-products). The participants did not drink, eat or smoke within 30 min of saliva sampling. In addition, we did not find any significant effect of cigarettes or alcohol consumption within 2 or 24 h. Another hypothesis is the impact of viral load. As we showed, the Ct values of nasopharyngeal RT-PCR were lower in true positive RT-LAMP samples than in false negative RT-LAMP samples (26 vs. 28), suggesting an impact of viral load on saliva RT-LAMP efficacy. However, when considering only high or significant SARS-CoV-2 loads in nasopharyngeal samples, saliva RT-LAMP sensitivity remained low. Thus, the viral load per se does not explain the weak performance of the assay.

We found that saliva RT-PCR identified more SARS-CoV-2 infections (9%) than nasopharyngeal RT-PCR (7%) and thus confirmed previous results suggesting that saliva RT-PCR could outperform nasopharyngeal RT-PCR. We recently showed that nasopharyngeal NAAT might be an imperfect reference method, which may contribute to increased estimates of sensitivity (and decreased specificity) for alternative tests^19^. When compared to a composite reference standard (including the results of nasopharyngeal RT-PCR and different RT-PCR tests on saliva), the sensitivity of saliva RT-PCR was higher than nasopharyngeal RT-PCR^19^.

Finally, our study confirmed the good performance of saliva RT-PCR and nasopharyngeal antigen testing as reliable alternative strategies to detect SARS-CoV-2 in both symptomatic and asymptomatic individuals in the ambulatory setting. Further work is needed to optimize an assay combining collected saliva and rapid point-of-care isothermal detection of SARS-CoV-2 RNA.

## Methods

### Study population and procedures

The institutional review board COMITE DE PROTECTION DES PERSONNES IDF 3 approved the study protocol prior to data collection (approval number 3840-NI) and all subsequent amendments. All stages of the study were carried out in accordance with relevant guidelines and regulations. Informed consent was obtained from all subjects or, if subjects were under 18, from a parent and/or parent or legal guardian . All adults and children, symptomatic or asymptomatic, referred to the two participating COVISAN centers in Paris, France were eligible, as described previously^19^. In accordance with the EasyCoV® assay (SkillCell-Alcen, Jarry, France) manufacturer instructions, the performances of saliva RT-LAMP were estimated on saliva tested in screening centers, immediately after collection (< 5 min) or stored immediately at 4 °C and then tested within an interval of no more than 90 min after collection. In addition, patients should have a valid nasopharyngeal SARS-CoV-2 RT-PCR test. Eligible persons received detailed oral and written information, adapted to their age. Data on sociodemographics, past medical history, presence of symptoms, consumption of alcohol, coffee, food, smoking and tooth brushing in the hours before testing were collected. NPS samples were sent to the Assistance Publique Hôpitaux de Paris APHP high throughput platform for RT-PCR testing as part of routine care (reference method). Participants were asked to self-collect saliva samples after after swishing saliva around in their mouths for 30 s. Saliva samples were tested directly in the screening center (see below) and then centralized for RT-PCR testing and frozen at -80 °C within 24 h.

### Virology methods

#### Nasopharyngeal RT-PCR

NPS samples were centralized and processed according to the routine procedure^19^ (appendix). Nucleic acid extraction was performed with MGIEasy® Nucleic Acid Extraction Kit (MGI Tech Co, Shenzhen, China) on an MGISP-960 instrument (MGI Tech Co). SARS-CoV-2 RNA amplification was done using the TaqPath™ COVID-19 CE IVD RT PCR Kit (Thermo Fisher Scientific, Coutaboeuf, France). The technique provides results expressed as a cycle threshold (Ct) for each gene target (ORF1ab, N and S genes). The cutoff value of the RT-PCR Ct used to distinguish high/significant and moderate/low SARS-CoV-2 loads with the TaqPath™ COVID-19 CE IVD RT PCR Kit was 28^29^. Ct values greater than or equal to 32 corresponded to low viral loads.

#### Saliva RT-PCR

Saliva samples were tested at the APHP high throughput platform with RT-PCR on MGI instruments as described above^19^ (appendix). A 300 µl aliquot of saliva was mixed with 300 µl of NucliSENS® lysis buffer (Biomerieux, Marcy l'Etoile, France). Nucleic acid extraction and SARS-CoV-2 RNA amplification were performed with the same procedure used for nasopharyngeal RT-PCR.

#### Saliva RT-LAMP

The EasyCov® (SkillCell-Alcen, Jarry, France) test is a CE-marked extraction-free RT-LAMP test specifically developed for saliva samples at the point of care (saliva POC-LAMP). Detection of SARS-CoV-2 was carried out according to the manufacturer’s instructions (appendix). The procedure includes one step involving virus inactivation and lysis at 80 °C for 10 min and another step involving viral genome amplification at 65 °C for 30 min. The two steps take place in the Easyvid® system. After amplification, a reagent sensitive to pH is added to reveal the amplification. The result is immediately read by visual observation. The color turns yellow for a sample positive for SARS-CoV-2 RNA and remains orange for a sample negative for SARS-CoV-2 RNA.

#### Nasopharyngeal rapid antigen test

Nasopharyngeal Ag testing was performed with the Standard Q COVID-19 Ag test (SD Biosensor®, Chuncheongbuk-do, Republic of Korea). Standard Q COVID-19 Ag test is a chromatographic immunoassay for the detection of the SARS-CoV-2 nucleocapsid (N) antigen. The result was read after 15 to 30 min according to the manufacturer’s instructions.

### Statistical analysis

Sample size was calculated assuming that the sensitivity of the index tests was greater than or equal to 60%. To allow sufficient precision (± 10%), 93 subjects with positive nasopharyngeal RT-PCR were needed in each of the two subgroups (symptomatic and asymptomatic participants). As preliminary results indicate that viral loads were not different between symptomatic and asymptomatic patients, the scientific committee of the study, during a planned meeting on December 16, 2020, recommended performing the analysis as soon as 93 subjects with positive nasopharyngeal RT-PCR were included, whether symptomatic or asymptomatic.

RT-PCR results were considered positive if at least one gene was detected. Analyses of tests results were carried out blind to the result of the others and ti the participant’s clinical data. For the RT-PCR technique, the Ct values reported are those for the ORF1a gene, and if not amplified, of the N gene (and of the S gene if the N gene was not amplified).

Quantitative data were expressed as medians [interquartile range], and qualitative data as numbers (percentages). The diagnostic accuracy of the index tests was evaluated by calculating sensitivity and specificity. Confidence intervals were calculated by the exact binomial method. Subgroup analyses were performed according to (1) the presence of symptoms on the day of testing, (2) the Ct value of the nasopharyngeal RT-PCR, expressed as low (at least one of the 3 targets with Ct ≤ 28, i.e. high viral shedding), or high (all 3 targets with Ct > 28, i.e. low viral shedding) and (3) the consumption of alcohol, coffee, food and smoking or tooth brushing before sample collection.

Sensitivity analyses were performed considering 6 alternate criteria for positivity for the reference standard: (1) ≥ 2 positive targets with nasopharyngeal RT-PCR, (2) ≥ 1 positive target with nasopharyngeal RT-PCR and at least one of the 3 targets with Ct < 32, (3) ≥ 1 positive target with saliva RT-PCR, (4) ≥ 1 positive target with either the nasopharyngeal or saliva RT-PCR, (5) ≥ 1 positive target with either the nasopharyngeal or saliva RT-PCR and at least one of the 3 targets with Ct < 32, and (6) NPS antigen test.

Quantitative variables were compared with Wilcoxon's test, with a significance level of 5%. The statistical analysis was performed using R software (http://cran.r-project.org/). The reporting of results followed the Standards for Reporting Diagnostic accuracy studies (STARD 2015) guideline^30^.

### Role of the funding sources

The funding sources had no role in the study’s design, conduct and reporting.

## Supplementary Information

Supplementary Information.
