# Supplementary material for: Evaluation of a saliva molecular point of care for the detection of SARS-CoV-2 in ambulatory care
Source: Sci Rep. 2021 Oct 26;11:21126. doi: 10.1038/s41598-021-00560-8 (PMC8548486; doi:10.1038/s41598-021-00560-8)
Supplement: Supplementary file 1 — Supplementary Information. [file 41598_2021_560_MOESM1_ESM.docx]

**Appendix**

**SARS-CoV-2 RT-PCR on nasopharyngeal samples**

NPS were inactivated at 56°C for 30 minutes. Nucleic acid extraction was performed on 180 µL of NPS with MGIEasy Nucleic Acid Extraction Kit (MGI Tech Co, Shenzhen, China) on a MGISP-960 instrument (MGI Tech Co). SARS-CoV-2 RNA amplification was done using TaqPath™ COVID 19 CE IVD RT PCR Kit (Thermo Fisher Scientific, Coutaboeuf, France). This test is a multiplex real-time RT-PCR test intended for the qualitative detection of nucleic acid from SARS-CoV-2. The kit contains three primer/probe sets specific to three different SARS-CoV-2 genomic regions (ORF1ab, N and S-genes) and primers/probes for bacteriophage MS2 used as internal control of amplification and extraction. A minimum of one negative control and one positive control was use for each run. Dilution of inactivated SARS-CoV-2 cell-culture supernatant (provided by Virology laboratory, Hospices Civils de Lyon) was added once a day in each production line as for external control. The technique provides results expressed as a cycle threshold (Ct) for each gene target.

**SARS-CoV-2 RT-PCR on saliva samples**

Saliva aliquots, stored frozen at minus 80°C, were thawed, equilibrated to room-temperature and then homogenized with a vortex for ﬁve seconds, and the 300 µl was mixed with 300 µl of NucliSENS® lysis buffer (Biomerieux, Marcy l'Etoile, France) and then extracted with the same procedure used for the nasopharyngeal samples. Saliva nucleic acids extracts were tested with the same RT-PCR procedure than for SARS-CoV-2 RT-PCR on nasopharyngeal samples.

**RT-LAMP assay on saliva samples**

The test EasyCov® (SkillCell-Alcen, Jarry, France) is a CE-marked extraction-free RT-LAMP test specifically developed for saliva samples. Detection of SARS-CoV-2 was carried out according manufacturer’s instructions (EasyCOV®, SkillCell) (appendix). In each screening center, saliva samples were tested immediately after collection (<5 minute) or stored immediately at 4°C and then tested within a maximal 90 minutes interval after collection. Briefly, 200 μl of saliva was mixed with the pretreatment buffer in tube 1 placed into the Easyvid® system for automated inactivation and lysis step (heating at 80 ° C for 10 minutes). Tube 1 is then taken out of Easyvid® and left to stand for 1 minute at room temperature. Three microliters of pretreated saliva sample are introduced into tube 2 containing RT-LAMP reaction mix. Tube 2 is incubated at 65 ° C for 30 minutes in dedicated positions of the Easyvid® system for viral RNA amplification. Once the 30 minutes have elapsed, tube 2 is taken out of the Easyvid® system and left to stand for 1 minute at room temperature. One microliter of revelation reagent is introduced in tube 2. The result is immediately read by visual observation. The color turns yellow for a sample positive for SARS-CoV2 RNA and remains orange for a sample negative for SARS-CoV2 RNA.
